# Emergency General Surgery: evolution of a subspecialty by stealth

**DOI:** 10.1186/s13017-015-0058-x

**Published:** 2016-01-04

**Authors:** L. Pearce, S. R. Smith, E. Parkin, C. Hall, J. Kennedy, A. Macdonald

**Affiliations:** Clinical Research Fellow, Department of General Surgery, Central Manchester University Hospitals Foundation Trust, Oxford Road, Manchester, M13 9WL UK

## Abstract

**Background:**

Emergency surgical patients account for around half of all NHS surgical workload and 80 % of surgical deaths. Few trainees opt to CCT in General Surgery, and there is no recognised subspecialty training program in Emergency General Surgery (EGS). Despite this lack of training and relevant assessment by examination, there appears to be an increasing number of EGS posts advertised. This study aims to provide information about potential future employment opportunities for surgical trainees.

**Methods:**

All consultant surgeon posts, advertised in the British Medical Journal between January 2009 and December 2014 were included. Data collected included specialty, region and institute of advertised post. For the purposes of statistical analysis, data was divided into two separate year bands: 2009–2011 and 2012–2014. Statistical analysis was by Chi-squared test; *p* <0.01 was considered statistically significant. An online tool was also used to determine experience and attitudes towards EGS amongst Consultant members of the ASGBI and all UK trainees in national training number (NTN) posts.

**Results:**

Over the six-year study period, there were 1240 consultant job adverts in a general surgical specialty. Nine hundred and 75 were substantive posts; the region with the most jobs was London and the South East (*n* = 278). There were 55 jobs advertised in EGS, either with (20) or without (35) another subspecialty. The number of EGS adverts increased significantly in 2012–14 compared to 2009–11 (*p* = 0.008). 229 (28 %) Consultants and 309 (22 %) trainees responded to the survey. 16 % of consultants work in NHS institutions with Emergency General Surgeons. Only 21 % of trainees believe EGS will be delivered by EGS consultants in the future whilst 8.2 % of trainees stated EGS as their career plan. Less than half of all UK consultant surgeons see EGS as a subspecialty.

**Conclusions:**

This data demonstrates increasing societal need for EGS consultants over the last six years and the emergence of Emergency Surgery as a new subspecialty. In order to meet the EGS needs of the NHS, general surgical training and the examination system need to be revised.

## Background

Centralisation of services within the UK’s National Health Service (NHS) has led to increasing subspecialisation within general surgery over the last ten years. Whilst this may be considered beneficial for elective care, numbers of surgeons with the diverse skill set required for the Emergency General Surgery (EGS) workload (50 % of all surgical workload, 80 % of surgical deaths) is reducing. More recently, through the publication of key documents and statements [1–5], there has been an increase in focus on Emergency General Surgical patients and the acute care surgical services that institutions are able to provide to manage them. However, for subspecialists, general surgical emergencies (often outside of their trained subspecialty) continue to be a major part of their consultant practice [6].

Standards have been set for the provision of emergency surgical care in the UK [2]. Senior prompt decision making has been shown to improve emergency surgical patient experience and outcomes [7, 8]. Consultant leadership is essential to ensure the safety of patients managed by the relatively new concept of ambulatory emergency care services within surgery [9].

Several different models of acute surgical service provision have been implemented by NHS institutions across the UK. Some larger centres provide simultaneous upper gastrointestinal, lower gastrointestinal and hepatobiliary daytime and ‘out of hours’ acute service. However, this model of care reduces subspecialist availability for elective work (rewarded by NHS tariffs) and is not possible in hospitals with small numbers of subspecialty consultants.

Many centres have appointed Consultant Emergency General Surgeons to provide a consultant led and consultant delivered acute care surgical service for emergency patients. As such, the term ‘Emergency General Surgeon’ is now one with which we are becoming increasing familiar in the UK. However, EGS remains an unrecognised UK training subspecialty without a separate higher surgical training programme [10] or modular competency based assessment.

## Aims

This study aims to ascertain UK Consultant and Trainees experiences and attitudes towards Emergency General Surgery and to provide information about potential future employment opportunities for trainees.

## Methods

An online survey tool was used (www.surveymonkey.net) to determine UK experience and attitudes towards Emergency General Surgery (EGS). Two separate online links were sent to consultant members of the ASGBI and all UK trainees in national training number (NTN) posts.

A review of all consultant general surgeon posts advertised in the British Medical Journal (BMJ) between 1^st^ January 2009 and 31^st^ December 2014 (72 months) was undertaken. Posts in general surgical specialities were included. Advertisements for plastic surgery, orthopaedic, cardiothoracic, urology, otorhinolaryngology, academic posts, senior lecturer and locum agency posts were excluded.

Job adverts were categorised as follows:

- Academic
- Breast
- Breast and general surgery
- Colorectal
- Colorectal and general surgery
- EGS/EGS and general surgery
- EGS and another subspecialty
- Endocrine
- Endocrine and general surgery
- General only
- Hepatobiliary
- Hepatobiliary and general surgery
- Remote and rural surgery
- Transplant
- Trauma
- Upper gastrointestinal
- Upper gastrointestinal and general surgery
- Vascular
- Vascular & general surgery
- Other

Data was also collected on the region, term and basis of appointment (locum or substantive, full or part time) and specialty advertised.

All analyses were performed using Stata, version 11.2 (College Station, TX, USA). We divided the data up into two discrete year bands – 2009–11 & 2012–14 – and explored how job adverts had changed over time using the Chi-squared test. *P* <0.01 was considered statistically significant.

## Results

Whilst 44 % of consultants surveyed view EGS as a subspecialty, a minority of consultant surgeons surveyed (16 %) currently work in institutions with EGS consultants. More recently appointed consultants less often felt ready for independent EGS duty than those appointed previously (10–20 years in post; 80 % ready compared with 2–5 years in post; 62 % ready). More than half (52 %) of consultants in post for less than five years would have felt ‘better’ prepared’ for EGS with a dedicated period of EGS training, compared with 13 % of those in post for less than twenty years (*p* = 0.022). Nearly all trainees (92.4 %) felt more intensive training in emergency surgery would be beneficial to their general surgical training i.e. 6-month post in acute surgery. Seventy per cent of Consultant surgeons envisage a future model of EGS cover utilising EGS surgeons compared with 21 % of trainees.

There were 1240 consultant jobs in a general surgical specialty advertised in the BMJ over the 6-year study period. Nine hundred and 75 were substantive posts. Eighty nine per cent of all posts (*n* = 1104) were advertised on a full time basis with less than full time considered in ten percent of posts (*n* = 124). Only one percent of all posts were advertised on a purely part time basis (80 % breast subspecialty).

The region with the most jobs was London and the South East (*n* = 278) (see Table 1). The total number of posts was 645 in 2009–11 and 595 in 2012–14. Table 2 displays job adverts according to subspecialty, comparing 2009–11 with 2012–14.

**Table 1:** Regional consultant surgeon job advertisements in the British Medical Journal 2009–2014

Region	Number of posts advertised
North West	155
North East	13
North & Yorkshire	136
West Midlands	98
East Midlands	73
Eastern	95
London	115
South West	106
South East	163
Scotland	191
Wales	52
N. Ireland	43
Total	1240

**Table 2:** Consultant surgeon job advertisements in the British Medical Journal 2009–2014

Specialty	2009–2011	2012–2014	Overall
Academic	1	1	2
Breast	104	94	198
Breast & general	34	16	50
Colorectal	94	98	192
Colorectal & general	69	52	121
EGS/EGS & general	11	24	35
EGS & other specialty	8	12	20
Endocrine	2	2	4
Endocrine & general	1	2	3
General only	79	91	170
HPB	24	18	42
HPB & general	1	3	4
Remote & rural	4	0	4
Transplant	18	24	42
Trauma	2	2	4
UGI	61	44	105
UGI & general	50	52	102
Vascular	70	55	125
Vascular & general	10	4	14
Other	2	1	3
Total	645	595	1240

In total, there were 55 jobs advertised in EGS, either with (20) or without (35) another subspecialty. The number of EGS adverts increased significantly in 2012–14 compared to 2009–11 (*p* = 0.008). London and the South East were the highest recruiters to EGS posts (10 % of all consultant surgeon posts advertised in the region). There was a borderline decrease in the number of breast & general jobs advertised (*p* = 0.021). Numbers of vascular & general jobs and purely general jobs did not change significantly over time.

## Discussion

It is an exciting time for Emergency General Surgery in the UK and changes should be embraced. Our study has highlighted the disparity between public and societal need, and the career aspirations of UK higher surgical trainees.

We have demonstrated evidence of an expanding national acute surgical workforce in the form of Emergency General Surgeons. Apart from a borderline decrease in the number of breast & general jobs, recruitment to other subspecialties has not changed significantly over the past 6 years.

### International models of care and the provision of training

Focus upon elective subspecialisation, and subsequent centralisation of services, has resulted in near extinction of the traditional “general surgeon” who regularly operated on multiple organ systems. There is worldwide debate surrounding the provision of acute care surgical services [11], appropriate models of emergency surgical care provision and related issues of decreased training time, operative exposure and ever increasing “super-subspecialisation” [12].

In recent years, international surgical colleges have published standards for emergency surgical care [2, 13, 14], but there remains abundant multifactorial heterogeneity between institutional models facilitating acute care surgical service provision [11]. The development of the specialty of Acute Care Surgery (ACS), Trauma and Critical Care in the USA has provided dedicated acute care subspecialty training for surgical residents. Whilst the training models are varied as dictated by widespread geographical variability in case mix (and particular trauma services) there is a minimum standard required within the curriculum for Acute Care Surgeons as detailed by the American Association for the Surgery of Trauma.

The Netherlands, Germany, Sweden and the UK are yet to develop nationally and establish a dedicated acute care surgical service [15]. Despite no UK national agreement on an appropriate acute model of care and a dedicated subspecialty emergency surgical training programme in the UK, our results show an increase in EGS appointments in the last 3 years. Similar appointments are being made in other European countries [16].

### Recent changes in UK Training

Surgical training has also become increasingly subspecialised. In 2013, vascular higher surgical training was uncoupled from general surgical training. Consequently, future vascular surgeons will not be adequately trained to provide acute emergency general surgical care at consultant level. Furthermore, few trainees opt to pursue a Completion Certificate of Training (CCT) in general surgery and EGS (or ACS) is not a recognised subspecialty. Our study demonstrates significant increase in numbers of emergency general surgical consultant posts but lack of expansion of UK consultant subspecialty posts in the last 3 years. Trainees are therefore facing unpredictable employment opportunities at the end of higher surgical subspecialty training in the UK.

### Training in EGS

EGS is often perceived to be associated with unpredictable out of hours operating, ‘disruption’ to elective services and adverse outcomes by trainee participation in cases [7]. However, there is clear evident public and societal need for emergency general surgeons, which has been acknowledged by NHS organisations and Consultant surgeons. Despite this, UK higher surgical training programmes have not, in the most part, sought to address potential short falls in emergency general surgical training, and competencies or encouraged trainees to pursue a career in EGS. Numbers of index emergency procedures (i.e. laparotomies) at CCT fall short of the detailed clinical competencies, case mix and volume of cases required for CCT in other subspecialties. Emergency general surgical training remains acquired during out of hours service provision (limited by European Working Time Directive) on an ad hoc basis, is not standardised and a workforce gap has been created.

### Future of EGS

Hospitals in the UK and beyond are appointing more emergency general surgeons, but their exact role within an organisation and their level of surgical competence has not been clearly defined. Individuals appointed to these roles should be trained to make appropriate clinical decisions in the management of emergency general surgical patients and be able to perform complex emergency procedures. They will also need to enable trainees to acquire the necessary skill set for independent emergency surgical practice.

The increase in EGS posts demonstrated in our study highlights potential employment opportunities for future higher surgical trainees and supports the need for a dedicated modular or higher surgical training programme in EGS. As not all emergency surgical skills can be developed in the elective setting, a dedicated period of emergency surgical training and assessment during higher surgical training could be beneficial by facilitating exposure to an array of complex and common emergency cases within the constraints of EWTD.

## Conclusion

It is likely that EGS provision in the UK NHS will continue to undergo significant change over the next decade. EGS is emerging as a subspecialty in its own right and trainees should consider future employment opportunities when declaring chosen subspecialty training. The Higher Surgical Training programme and examination system in general surgery should be modified to address these changes to ensure emergency surgical patients in the NHS receive expeditious, high quality care.
